# Interleukin-17A contributes to the development of post-operative atrial fibrillation by regulating inflammation and fibrosis in rats with sterile pericarditis

**DOI:** 10.3892/ijmm.2015.2204

**Published:** 2015-05-08

**Authors:** XIAO-XING FU, NING ZHAO, QIAN DONG, LI-LI DU, XIAO-JUN CHEN, QIONG-FENG WU, XIANG CHENG, YI-MEI DU, YU-HUA LIAO

**Affiliations:** 1 Research Center of Ion Channelopathy, Institute of Cardiology, Union Hospital, Tongji Medical College, Huazhong University of Science and Technology, Wuhan, Hubei 430022, P.R. China; 2 Emergency Department, Central Hospital of Wuhan, Wuhan, Hubei 430014, P.R. China; 3 Fujian University of Traditional Chinese Medicine, Fuzhou, Fujian 350108, P.R. China

**Keywords:** interleukin-17A, atrial fibrillation, inflammation, fibrosis, sterile pericarditis

## Abstract

Post-operative atrial fibrillation (AF) remains a common cause of morbidity. Increasing evidence indicates that inflammation and atrial fibrosis contribute to the pathogenesis of this condition. Interleukin (IL)-17A, a potent pro-inflammatory cytokine, has been implicated in the development of a number of cardiovascular diseases. However, its role in post-operative AF remains unknown. In the present study, sterile pericarditis (SP) was induced in rats by the epicardial application of sterile talc. AF was induced by transesophageal burst pacing. Western blot analysis was applied to quantify the expression of IL-17A. Quantitative PCR was used to detect the mRNA expression of IL-17A, IL-6, IL-1β, transforming growth factor-β1 (TGF-β1), collagen type 1 (Col-1), collagen type 3 (Col-3) and α-smooth muscle actin (α-SMA). Gelatin zymography and reverse gelatin zymography were used to quantify the levels of matrix metalloproteinases (MMPs) and tissue inhibitors of MMPs (TIMPs). Histological analyses were performed to determine the extent of tissue inflammation and fibrosis. The rats with SP presented with a shorter refractoriness, a higher incidence and duration of AF, an enhanced susceptibility to developing AF, increased mRNA levels of AF-related pro-inflammatory cytokines (IL-6, IL-1β and TGF-β1), as well as marked atrial inflammation and fibrosis. The atrial IL-17A levels were elevated and correlated with the probability of developing AF. Treatment with anti-IL-17A monoclonal antibody decreased the levels of atrial IL-17A, prolonged refraction and markedly suppressed the development of AF. Simultaneously, inflammation and fibrosis were alleviated, which was further demonstrated by a decreased expression of AF-related pro-inflammatory cytokines, a down-regulation in fibrosis-related mRNA expression (Col-1, Col-3 and α-SMA) and by the decreased activity of MMP-2/9 and TIMPs. Thus, the findings of our study indicate that IL-17A may play a pathogenic role in post-operative AF by inducing inflammation and fibrosis in rats with SP.

## Introduction

Atrial fibrillation (AF) is the most frequent complication following cardiac surgery, affecting 10–65% of patients. Postoperative AF can be life threatening and is associated with a significant morbidity and prolonged hospital stay (1). However, the exact pathophysiological mechanisms responsible for the onset and perpetuation of post-operative AF are not yet completely understood. Post-operative AF appears to be associated with inflammation, fibrosis and oxidative stress (2).

Interleukin (IL)-17A, a pleiotropic pro-inflammatory cytokine, has been implicated in the development of numerous inflammatory reactions (3,4). Previous studies have demonstrated that IL-17A stimulates a variety of cells to release pro-inflammatory cytokines [IL-1β, IL-6 and transforming growth factor-β1 (TGF-β)] (5), while also synergizing with other cytokines, such as IL-1β, IL-6, interferon-γ (IFN-γ) and tumor necrosis factor-α (TNF-α) to enhance pro-inflammatory responses (6,7). In addition, IL-17A induces cardiomyocyte apoptosis (8,9) and enhances the production of collagens and matrix metalloproteinases (MMPs) (10,11), suggesting that IL-17A plays a role in myocardial remodeling and fibrosis (9,12). The enhanced expression of IL-17A has been reported in several cardiovascular diseases, including atherosclerosis (13), myocarditis (14,15), dilated cardiomyopathy (16) and myocardial ischemia/reperfusion injury (8,17). However, to the best of our knowledge, there are limited data available to date on the role of IL-17A in post-operative AF.

The model of sterile pericarditis (SP) is used to mimic many of the clinical symptoms following heart surgery and has thus proved to be useful in the study of post-operative atrial arrhythmia (18). The present study was designed to: i) to assess the changes in IL-17A expression, atrial inflammatory responses, fibrosis, along with arrhythmia vulnerability induced by transesophageal burst pacing in rats with SP and ii) to determine whether the blockade of IL-17A prevents AF by inhibiting inflammation and fibrosis in rats with SP.

## Materials and methods

### Animal

Sprague-Dawley rats weighing 250±25 g were purchased from the Laboratory Animal Center of Huazhong University of Science and Technology (Wuhan, China). All the animals were kept in a pathogen-free environment at the Experimental Animal Center of Tongji Medical College of Huazhong University of Science and Technology. The study was approved by the University Animal Ethics Committee in accordance with the guidelines established in the Guide for the Care and Use of Laboratory Animals (NIH Publication, revised 2011).

### Creation of the model of SP

SP was created as previously described (18) under sterile surgical conditions. Briefly, the rats were anesthetized with pentobarbital sodium (40 mg/kg), orally intubated and connected to a rodent ventilator. The pericardial sac was exposed through a left thoracotomy in the second intercostal space. The atrial surfaces were generously dusted with talcum powder and a single layer of gauze was placed on the left and right atrial free walls. The chest was then closed in standard fashion. The animals were administered antibiotics (penicillin) and analgesic agents (buprenorphine) and were then allowed to recover. The sham-operated animals were subjected to the same procedure without a pericardiotomy. At the end of the experiments, the rats were re-anesthetized and the hearts were excised and immersed into 4% paraformaldehyde or snap-frozen at −80°C for further analysis.

### Treatment

The rats were injected intraperitoneally with 100 *µ*g anti-rat IL-17 monoclonal antibody (mAb; clone eBio17B7, IgG2a) or 100 *µ*g rat IgG2a isotype control mAb (both from eBioscience, San Diego, CA, USA) 5 min prior to pericardiotomy. The antibody injection was repeated every 48 h.

### Post-operative electrophysiological analysis

On the fourth post-operative day, the rats were re-anesthetized with pentobarbital sodium (40 mg/kg). The surface electrocardiograms (ECG; lead II and aVR) were recorded continuously during the experiment. A clinically available 6-French 10-pole coronary sinus electrodes catheter, (ten 0.5 mm circular electrodes; inter-electrode distance, 2.0 mm; electrode pair spacing, 6.0 mm), was inserted into the esophagus and positioned at a site at which the lowest threshold could capture the atrium. Atrial pacing was performed at the fourth diastolic threshold, through the distal electrodes pair of the catheter using an electrical simulator with S1S1 stimulus cycle lengths (CLs) down to a duration of 10 msec (Model YC-2; Chengdu Instrument Factory, Chengdu, China).

Regular pacing and standard S1S2 pacing protocols were used to determine the standard electrophysiological parameters, Wenckebach periodicity (WP), sinus node recovery time (SNRT), rate corrected SNRT (SACT) and atrial and atrioventricular (AV) nodal refractory periods (ARPs and AVNRPs). To induce AF, 5 consecutive bursts of rapid stimulation (25, 30, 40, 50 and 83 Hz) for 30 sec were implemented with a 5-min interval. AF was defined as rapid and fragmented atrial electrograms with an irregular ventricular rhythm for at least 1 sec immediately following the burst pacing. The number, duration and probability of inducible AF episodes were analyzed.

### Hematoxylin and eosin (H&E) and Masson’s staining

Tissue samples obtained from the atria were fixed with 4% paraformaldehyde, embedded in paraffin and sliced into 4-*µ*m-thick sections. The sections were stained with H&E and Masson’s trichrome. The analysis of the images at x400 magnification (15–20 images from 3–5 sections) was performed using Image-Pro 6.2 software. The analyses were performed by at least 2 independent investigators on coded specimens in a blinded manner.

### Quantitative PCR (qPCR)

qPCR was performed as previously described (19,20) using gene-specific primer pairs (Table I). In all the experiments, negative controls were applied in the absence of the reverse transcriptase reaction and glyceraldehyde 3-phosphate dehydrogenase (GAPDH) was used as a control. The relative expression quantity 2^−ΔΔCt^ value was calculated to compare the differences among groups. The result for each gene was obtained from 3 independent measurements (n=4/group) performed in duplicate.

### Western blot analysis

Protein-extracts of snap-frozen left atrial tissue samples were prepared according to standard procedures. The protein concentrations in the supernatants were measured using a BCA kit (Pierce, Rockford, IL, USA). Samples containing 80 *µ*g of total protein were boiled in SDS loading buffer, separated on a 12% sodium dodecyl sulfate-polyacrylamide gel (SDS-PAGE) gel, and transferred onto nitrocellulose membranes. After blocking, the membranes were incubated with IL-17A antibody (#212-401-B32; Rock land Immunochemicals Inc., Gilbertsville, PA, USA) diluted at 1:1,000 and then incubated successionally with secondary horseradish peroxidase-conjugated anti-rabbit IgG antibody (Proteintech Group, Chicago, IL, USA). Proteins were visualized using an enhanced chemiluminescence kit (Pierce). The intensity of the β-actin (1:1,000; Abcam, Cambridge, MA, USA) band was used as a loading control for the comparison between samples, as previously described (21).

### Gelatin zymography

The gelatinolytic activities of MMP-2 and MMP-9 were detected by gelatin zymography as previously described in detail (22). Briefly, the atria supernatants (containing 20 *µ*g proteins/lane) were separated by electrophoresis on a 12% SDS-PAGE gel impregnated with 0.1 mg/ml of gelatin (Sigma, St. Louis, MO, USA) as a substrate under non-reducing conditions (0.125 M Tris-HCl, pH 6.8, 4% SDS, 20% glycerol and 0.06% bromophenol blue). Following electrophoresis, the gel was washed twice for 40 min in renaturing buffer (2.5% Triton X-100) with gentle agitation at room temperature to remove the SDS, and then incubated at 37°C overnight in zymogram developing buffer (50 mM Tris-HCl, pH 7.6, 200 mM NaCl, 5 mM CaCl_2_, 1 *µ*M ZnCl_2_ and 0.02% Brij-35). After staining with Coomassie brilliant blue, the gelatinase activities were identified as clear zones against a blue background. A MMP-2 positive control (Chemicon, Temecula, CA, USA) was loaded onto each gel as a standard to normalize the density values. Semi-quantitative densitometric analysis was carried out using the NIH program 1.62 and the results are expressed in arbitrary units.

### Reverse gelatin zymography

The activity of tissue inhibitors of MMPs (TIMPs) was analyzed by reverse zymography, as previoulsy described (23). Samples containing 20 *µ*g of atrial protein were separated by electrophoresis on a 12% SDS-PAGE gel prepared with 1 mg/ml gelatin and 0.1 mg/ml MMP-2. Following electrophoresis, the gels were washed twice with 2.5% Triton X-100 for 40 min at room temperature to remove the SDS. The gels were then incubated at 37°C for 48–72 h in 50 mM Tris-HCl and 10 mM CaCl_2_ at pH 7.6 and stained with 0.5% Coomassie brilliant blue, destained and scanned.

### Statistical analysis

All data are expressed as the means ± SD. Differences between groups were analyzed using a two-tailed Student’s t-test, analysis of variance (ANOVA), the Holm-Sidaks test, or the Chi-square test, where appropriate. Linear regression analysis was used to evaluate the association between the expression of IL-17A and the probability of inducible AF. A two-tailed P<0.05 was considered to indicate a statistically significant difference. All statistical analyses were performed using SPSS 13.0 software (SPSS, Inc. Chicago, IL, USA).

## Results

### Characterization of the model of SP

**Figure 1:**
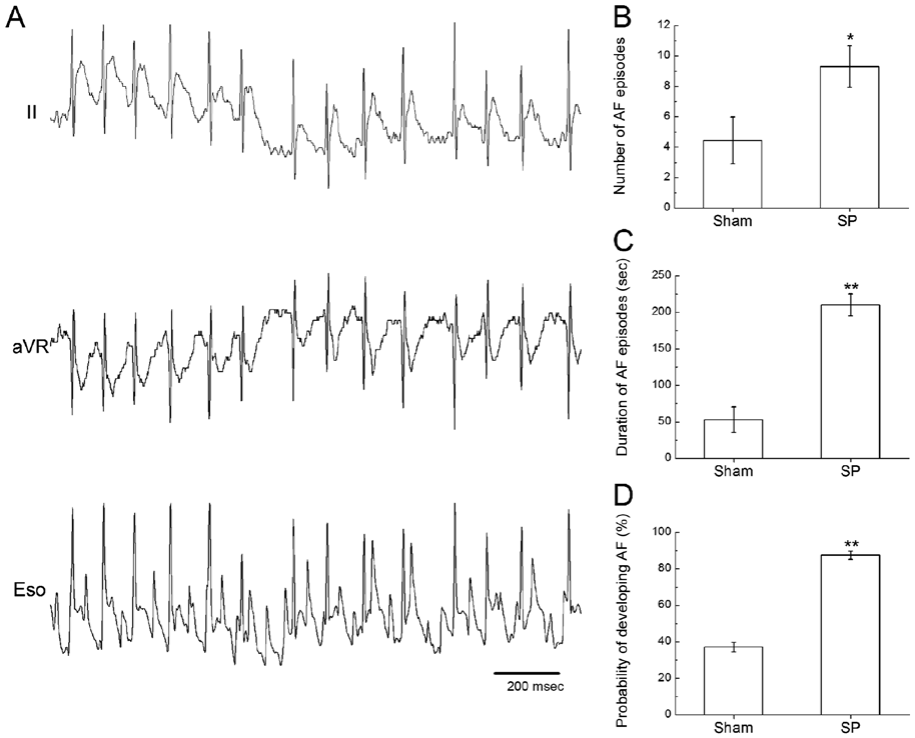
Analysis of the inducibility of atrial fibrillation (AF) in sham-operated rats (Sham) and rats with sterile pericarditis (SP). (A) Typical transesophageal and surface electrogram recordings following the induction of AF. (B) Quantification of the number of AF episodes. (C) Total duration of AF episodes. (D) Probability of the development of AF, defined as inducible episodes divided by the number of total testing maneuvers applied. ^*^P<0.05 and ^**^P<0.01 vs. Sham.

The basic cardiac electrophysiology data are presented in Table II. Both the ARPs and AVNRPs measured were significantly shorter in the rats with SP at the S1S1 CLs of 120, 110 and 100 msec with a 10 msec stepwise S1S2 reduction starting 10 msec after S1S1 (P<0.05). However, no significant differences were observed in the parameters examined. AF was repeatedly induced by burst pacing as described in the Materials and methods. Typical transesophageal and surface electrogram recordings following the induction of AF are illustrated in Fig. 1A. The rats with SP showed a higher susceptibility to the development of AF compared to the sham-operated rats, with a higher incidence (Fig. 1B) and duration of AF episodes (Fig. 1C), as well as an increased probability of developing AF (Fig. 1D).

**Figure 2:**
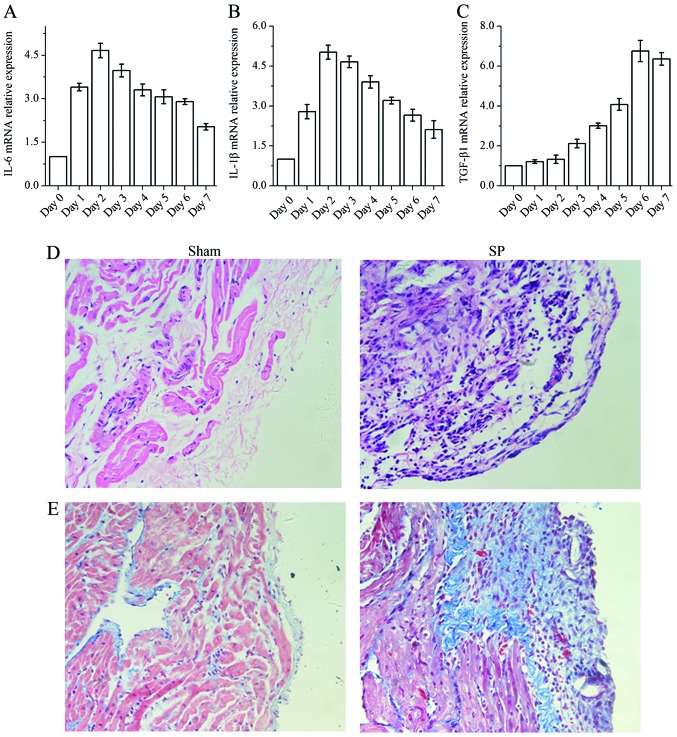
Assessment of inflammation and fibrosis in the sham-operated rats (Sham) and rats with sterile pericarditis (SP). (A–C) Relative mRNA level of atrial fibrillation (AF)-related pro-inflammatory cytokines: (A) interleukin (IL)-6, (B) IL-1β and (C) transforming growth factor-β1 (TGF-β1) in the atril samples during post-operative days 0–7. (D and E) Representative histological sections stained with (D) hematoxylin and eosin (H&E) and (E) Masson’s trichrome at 4 days after surgery. Original magnification, x400. The rats with SP presented with a significant number of infiltrating inflammatory cells and extensice fibrosis.

An increase in the expression of pro-inflammatory cytokines has been reported to be associated with post-operative AF (24,25) and in animal models of SP as well (26,27). Thus, in this study, we examined cytokine expression in the atria of the rats on post-operative days 0-7. The mRNA levels of IL-6 and IL-1β increased significantly as early as 1 day following pericardiotomy, reached peak levels on day 2 and then began to decrease, confirming a previous observation (27) (Fig. 2A and B). By contrast, the TGF-β1 mRNA level increased progressively (Fig. 2C).

Representative H&E stained and Masson’s trichrome stained atrial sections obtained from the sham-operated rats and the rats with SP are presented in Fig. 2D and E, respectively. The atrial myocytes from the sham-operated rats showed a normal composition of sarcomeres distributed throughout the cell, and the intracellular space also appeared normal. By contrast, the atrial myocytes of the rats with SP showed active perimyocarditis, which consisted of inflammatory infiltrate with lipid degeneration. Multiple inflammatory cell foci were evident in the myocardium. In addition, extensive interstitial fibrosis, as evidenced by Masson’s trichrome staining was observed in atrial samples from the rats with SP.

### IL-17A expression and induction of AF in rats with SP

**Figure 3:**
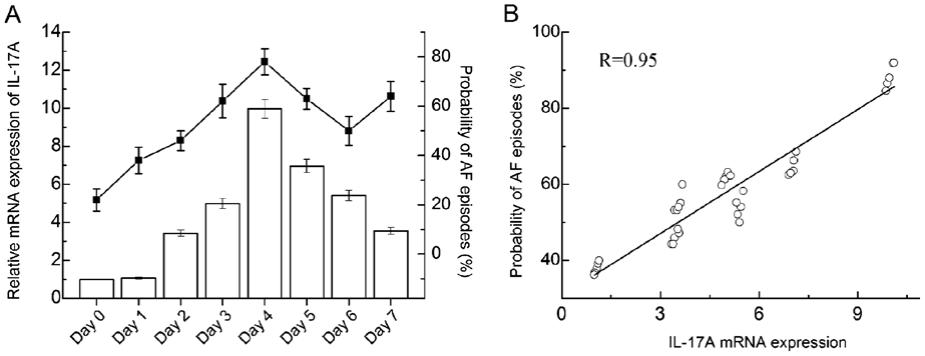
Inducibility of interleukin-17A (IL-17A) mRNA expression and atrial fibrillation (AF) in rats with sterile pericarditis (SP). (A) IL-17A mRNA expression and probability of developing AF during post-operative days 0–7. The time of the probability of developing AF coincided with the expression of IL-17A in the rats with SP. (B) Correlation between IL-17A expression and the probability of developing AF.

To determine the involvement of IL-17A in post-operative AF, we measured the IL-17A levels in the atrial samples following surgery. The mRNA expression of IL-17A began to increase at day 1 following surgery, reached a peak on day 4, and then gradually decreased (Fig. 3A). This result was confirmed by western blot analysis, which revealed an increased expression of IL-17A on day 4 compared with the sham-operated rats (P< 0.05; Fig. 6B). Of note, the time course of IL-17A expression coincided with the probability of AF episodes. When the level of IL-17A reached its peak on day 4, the probability of AF episodes increased to the highest level. Linear correlation analysis of the association between the expression of IL-17A and the probability of AF episodes was performed. The correlation coefficient was 0.95 (Fig. 3B). Our data indicate that IL-17A may contribute to the induction of AF in rats with SP.

### Neutralization of endogenous IL-17A reduces susceptibility to AF

**Figure 4:**
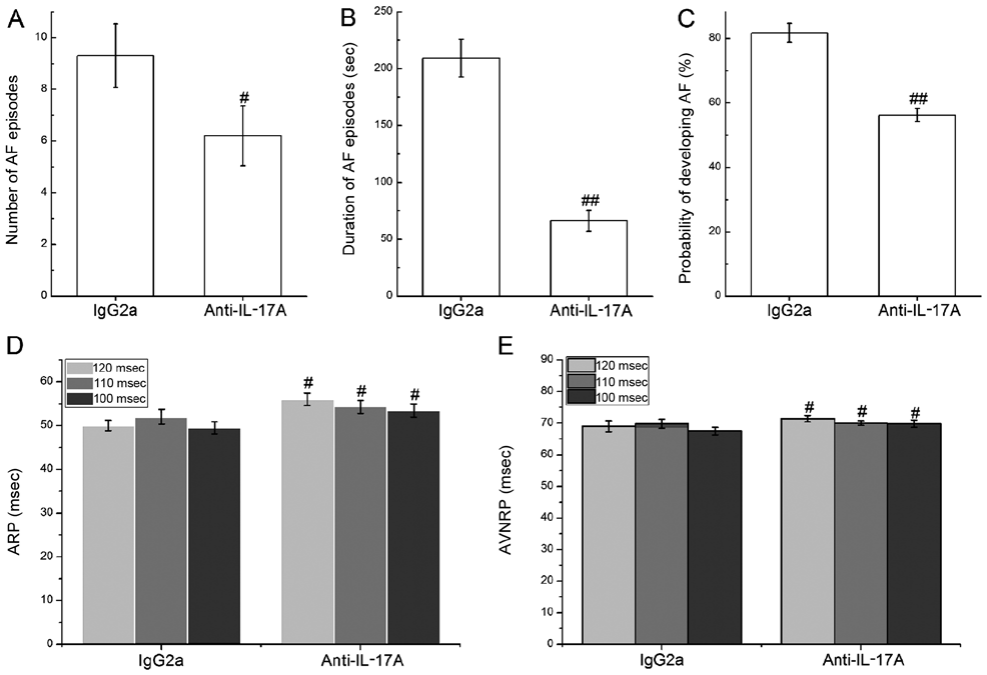
Effect of the neutralization of endogenous interleukin-17A (IL-17A) on the development of atrial fibrillation (AF) at 4 days after surgery in the rats with sterile pericarditis (SP). (A) Number of AF episodes. (B) Duration of AF episodes. (C) Probability of developing AF. (D) Atrial nodal refractory period (ARP). (E) Atrioventricular (AV) nodal refractory period (AVNRP). ^#^P<0.05 and ^##^P<0.01 vs. IgG2a control group.

To further investigate the role of IL-17A in post-operative AF, we treated the rats with SP systemically with neutralizing anti-IL-17A mAb 5 min prior to surgery. Our results revealed that the incidence and duration of AF episodes, as well as the probability of developing AF were significantly lower in the rats treated with anti-IL-17A mAb compared with those treated with the isotype control IgG2a (Fig. 4A–C). At the same time, treatment with anti-IL-17A mAb significantly increased the ARPs and AVNRPs to values comparable with those of the IgG2a control-treated group (Fig. 4D and E).

### Neutralization of endogenous IL-17A suppresses inflammation and fibrosis

**Figure 5:**
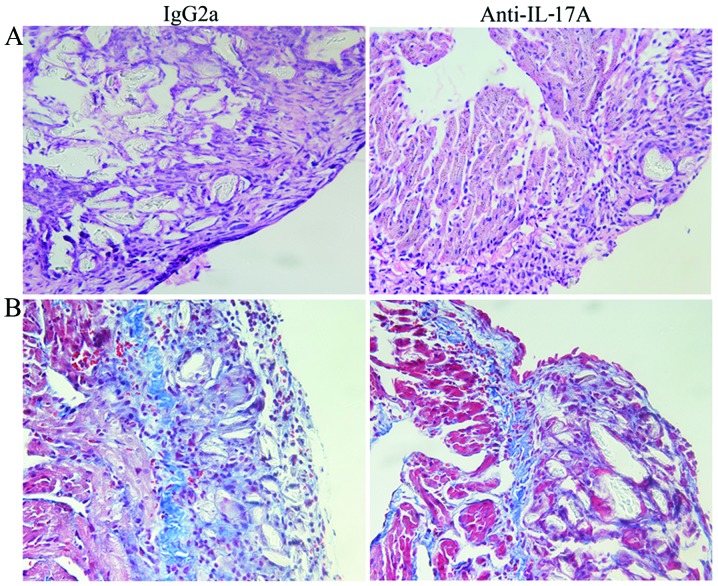
Effect of the neutralization of endogenous interleukin-17A (IL-17A) on inflammation and fibrosis in rats with sterile pericarditis (SP). Representative histological sections stained with (A) H&E and (B) Masson’s trichrome at 4 days after surgery. Original magnification, x400. Treatment with anti-IL-17A mAb resulted in a significant decrease in the number of infiltrating inflammatory cells and fibrosis.

**Figure 6:**
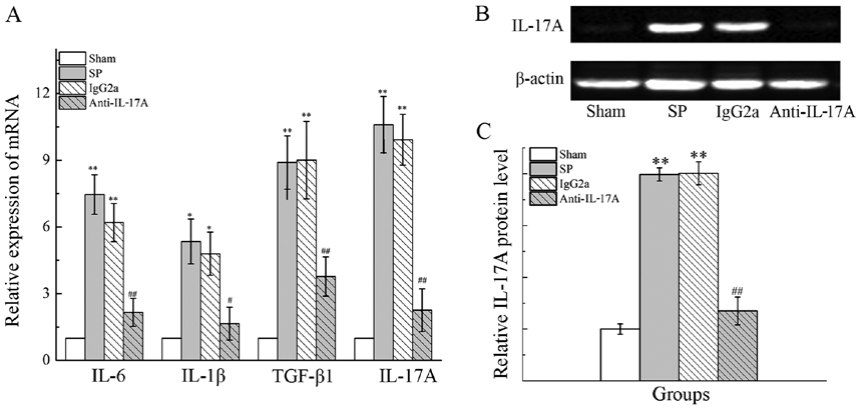
Expression of atrial fibrillation (AF)-related pro-inflammatory cytokines at 4 days after surgery. (A) Fold mRNA expression of interleukin (IL)-6, IL-1β, transforming growth factor-β1 (TGF-β1) and IL-17A. (B) Protein expression of IL-17A detected by western blot analysis. (C) Quantitative analysis of IL-17A protein expression. ^*^P<0.05 and ^**^P<0.01 vs. Sham. ^#^P<0.05 and ^##^P<0.01 vs. IgG2a. Sham, sham-operated rats; SP, rats with sterile pericarditis.

**Figure 7:**
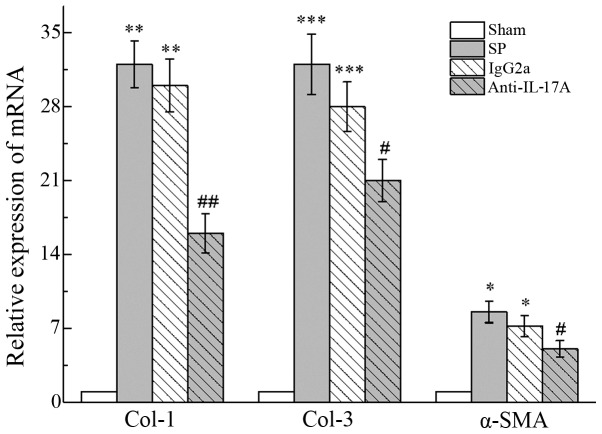
Relative mRNA level of collagen type 1 (Col-1), collagen type 3 (Col-3) and α-smooth muscle actin (α-SMA) in the atrial samples at 4 days after surgery. Data were normalized to the mRNA expression level of β-actin and are expressed relative to Sham. *P<0.05, ^**^P<0.01 and ^**^*P<0.001 vs. Sham; ^#^P<0.05 and ^##^P<0.01 vs. IgG2a control. Sham, sham-operated rats; SP, rats with sterile pericarditis.

H&E staining revealed that treatment with the anti-IL-17A mAb resulted in a marked reduction in the infiltration of inflammatory cells into the atria compared to treatment with the isotype control IgG2a (Fig. 5A). Furthermore, the SP-induced increase in the mRNA expression of IL-6, IL-1β, TGF-β1 and IL-17A was significantly inhibited following treatment with anti-IL-17A mAb (Fig. 6A). The results obtained for IL-17A mRNA expression were confirmed at the protein level by western blot analysis and quantitative analysis (Fig. 6B and C). No changes were observed following treatment with the isotype contorl IgG2a. Notably, treatment with anti-IL-17A mAb markedly decreased the percentage area of fibrosis (Fig. 5B). Consistent with this observation, the mRNA expression of collagen type 1 (Col-1), collagen type 3 (Col-3) and α-smooth muscle actin (α-SMA), which indicate the activation of extracellular matrix (ECM) synthesis, was significantly downregulated following treatment with anti-IL-17A mAb (Fig. 7).

**Figure 8:**
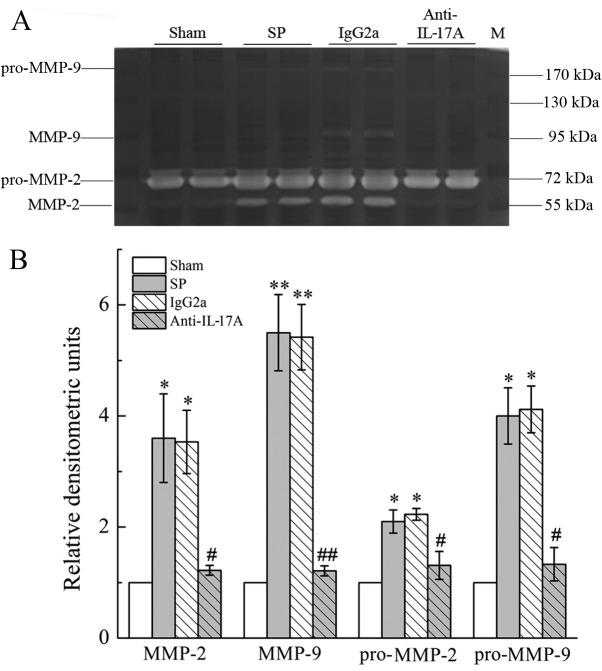
Changes in the activity of matrix metalloproteinase (MMP)-2 and MMP-3 in the atrial samples at 4 days after surgery. (A) A representative gelatin zymogram is shown. Gelatinolytic bands of 220, 95, 72 and 68 kDa corresponded to pro-MMP-9, active MMP-9, pro-MMP-2 and active MMP-2, respectively. Molecular mass markers (M) are indicated on right side. (B) Quantification of gelatinase activity was achieved by computer-assisted image analysis of the zymographic gels. The data are presented as the fold change vs. Sham. ^*^P<0.05 and ^**^P<0.01 vs. Sham; ^#^P<0.05 and ^##^P<0.01 vs. IgG2a control. Sham, sham-operated rats; SP, rats with sterile pericarditis.

**Figure 9:**
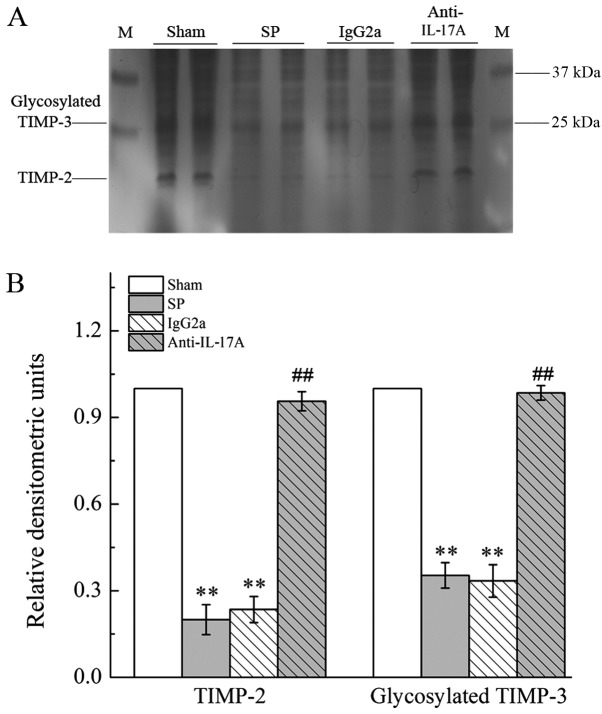
Changes in the activity of tissue inhibitors of MMPs (TIMPs) in the atrial samples at 4 days after surgery. (A) A representative reverse gelatin zymogram is shown. Dark bands indicate undigested gelatin stained with Coomassie blue, representing the zones of gelatinase inhibition. The migration positions of TIMP-2 and glycosylated TIMP-3 are indicated. Molecular mass markers (M) are indicated. (B) Quantification of reverse gelatin zymography. The data are presented as relative densitometric units.^**^P<0.01 vs. Sham; ^##^P<0.01 vs. IgG2a control. Sham, sham-operated rats; SP, rats with sterile pericarditis.

Imbalances in the levels of MMPs and TIMPs have been linked with AF (23,28). In this study, we used gelatinzymography to determine the activity of MMP-2 and MMP-9 in the atrial tissues.A representative gelatin zymogram is shown in Fig. 8A. Gelatinases of 220 and 95 kDa corresponded to pro-MMP-9 and an active form of MMP-9, respectively. Gelatinases of 72 and 68 kDa were considered to be pro-MMP-2 and its active form, respectively. Data analysis revealed significantly higher mean levels of pro-MMP-9, active MMP-9, pro-MMP-2 and active MMP-2 in the rats with SP compared with the sham-operated rats. However, their activities were significantly decreased following treatment with anti-IL-17A mAb (Fig. 8B). The activity of TIMPs was also determined by reverse gelatin zymography. Densitometric analysis of these TIMP activities revealed a significantly decreased TIMP-2 (19 kDa) and glycosylated TIMP-3 (22 kDa) activity in the rats with SP compared with the sham-operated rats. However, the activities of TIMPs returned to almost normal levels following treatment with anti-IL-17A mAb (Fig. 9).

## Discussion

In this study, we first characterized atrial pro-fibrillatory remodeling in rats with SP, observing that the expression levels of IL-17A correlated with the probability of developing AF. We then examined the role of IL-17A using anti-IL-17A neutralizing mAb. We found that the neutralization of endogenous IL-17A prevented the induction of AF, decreased inflammation and ameliorated the severity of atrial fibrosis in the rats with SP. Our results suggest that IL-17A contributes to the development of AF by regulating inflammation and fibrosis in rats with SP.

### Model of SP

SP occurs as a consequence of open-heart surgery, causing a range of comorbidities, including AF. In accordance with this fact, Pagé *et al* (18) developed the canine model of SP as an experimental counterpart to post-operative AF. However, owing to its small size, the low costs of its use and the fact that it is easy to handle, the rat has become an attractive mammalian model of AF (29–31). Therefore, in this study we induced SP in rats. AF was successfully induced by rapid transesophageal atrial pacing. The incidence of AF increased significantly on day 1 and peaked on day 4. In the rat model, we evaluated atrial structural remodeling and observed extensive inflammatory infiltrate and fibrosis, as well as the increased expression of AF-associated pro-inflammatory cytokines IL-6, IL-1β and TGF-β1 following surgery. We simultaneously evaluated the atrial electrophysiological properties, and found that both ARPs and AVNRPs were decreased in this model of SP. All these features were similar to those of SP in canines (18,27), which suggests that rats with SP are a useful model for the study of post-operative AF.

### IL-17A and AF

The pathogenesis of post-operative AF is multifactorial and involves a multitude of clinical and intraoperative factors. However, among the most important mechanisms responsible for post-operative AF are inflammatory changes occurring in the atrium within the first few days following cardiac surgery (2). Considering the pro-inflammatory effects of IL-17A, we investigated the role of IL-17A in post-operative AF using rats with SP. We observed the increased expression of IL-17A in the atrium beginning at day 1 following surgery, peaking on day 4 and decreasing thereafter in the rats with SP, which corresponded with the trend in the incidence of AF following surgery. Further linear analysis revealed that the expression of IL-17A positively correlated with the incidence of AF. Our results thus suggest that IL-17A contributes to the development of AF in rats with SP.

### IL-17A and inflammation/fibrosis

Extensive studies have demonstrated that IL-17A is a pro-inflammatory cytokine, as it induces the production of multiple cytokines and chemokines, which recruit neutrophils, macrophages and lymphocytes, thereby enhancing inflammation (3,4). IL-17A also stimulates the production of MMPs (10,11), promotes the expression of collagens and facilitates the proliferation and migration of cardiac fibroblasts (12). The pro-inflammatory and pro-fibrotic roles of IL-17A are further supported by the fact that the neutralization of IL-17 reduces the expression of the pro-inflammatory cytokines,IL-1β, IL-6 and TNF-α, in the heart, downregulating the expression of MMP-2/9, and thus ameliorating myocarditis-induced cardiac fibrosis, and therefore delaying the progression to dilated cardiomyopathy (16). In addition, in a recent study, Liao *et al* (8) suggested that the neutralization of IL-17A or the genetic abolition of IL-17A diminished neutrophil invasion and prevented myocardial ischemia-reperfusion injury.

In the present study, the neutralization of IL-17A with mAb significantly reduced the incidence of AF, as demonstrated by the reduced number and duration of AF episodes, as well as by the probability of AF induction in rats with SP. We also observed that the inhibition of IL-17A markedly decreased the atrial expression of IL-6, IL-1β, TGF-β and inflammatory cell recruitment. We further demonstrated that the blockade of IL-17A inhibited atrial fibrosis and decreased the level of Col-1, Col-3 and α-SMA in the rats with SP, similar to the effects reported in models of cardiac infarction (9,11) and myocarditis-induced cardiac fibrosis (16), and consistent with a potential role for IL-17A in cardiac fibrosis (9,10,12). In addition, we found that MMP-2 and MMP-9 activity decreased, while TIMP-2 and TIMP-3 activity increased in response to treatment with anti-IL-17A. Thus, IL-17A contributes to the development of AF by promoting inflammation and cardiac fibrosis in rats with SP.

### Study limitations

Due to species and etiology-specific considerations, our results should not be extrapolated directly to other models of AF or non-post-operative AF. Further human studies warranted to confirm our results. Although the 6-French catheter is an effective way to induce AF, is feasible to handle, and is durable for multiple use, its large surface area along with a relatively high direct current may not have precisely captured the right atrium of the small animal; thus this needs to be examined further.

In conclusion, in this study, we clearly demonstrate the significant increase in the expression of IL-17A in rats with SP, which may contribute to the development of AF by stimulating inflammatory responses and promoting cardiac fibrosis. Furthermore, neutralizing IL-17A attenuates the development and duration of AF in rats with SP. Our results thus indicate that IL-17A may be a novel target in the treatment of postoperative AF.

## Figures and Tables

**Table I:** Primers used for qPCR.

Gene	NCBI no.	Primer sequences (5′→3′)
GAPDH	NM_017008.4	Forward: GACATCAAGAAGGTGGTGAAGC
		Reverse: TGTCATTGAGAGCAATGCCAGC
IL-1β	NM_031512.2	Forward: CTCTGTGACTCGTGGGATGATG
		Reverse: CACTTGTTGGCTTATGTTCTGTCC
IL-6	NM_012589.2	Forward: AACGAAAGTCAACTCCATCTG
		Reverse: GGTATCCTCTGTGAAGTCTCC
TGF-β1	NM_021578.2	Forward: TGGCGTTACCTTGGTAACC
		Reverse: GGTGTTGAGCCCTTTCCAG
IL-17A	NM_001106897.1	Forward: ACAGTGAAGGCAGCGGTACT
		Reverse: GCTCAGAGTCCAGGGTGAAG
Col-1	NM _053304.1	Forward: GAGCGGAGAGTACTGGATCG
		Reverse: TACTCGAACTGGAATCCATC
Col-3	NM_032085.1	Forward: CAGCTGGCCTTCCTCAGACT
		Reverse: TGCTGTTTTTGCACTGGTATGTAA
α-SMA	NM_031004.2	Forward: CTGTGCTATGTCGCTCTGGA
		Reverse: ATAGGTGGTTTCGTGGATGC

PCR, polymerase chain reaction; GAPDH, glyceraldehyde 3-phosphate dehydrogenase; IL, interleukin; TGF-β1, transforming growth factor-β1; Col-1, collagen type 1; Col-3, collagen type 3; α-SMA, α-smooth muscle actin.

**Table II:** Evaluation of surface ECG parameters, transesophageal recording and atrial stimulation.

	Sham (n=10)	SP (n=17)
Heart beat (bpm)	450±24.5	433.8±21.6
SCL (msec)	133.8±7.4	138.5±6.9
PR interval (msec)	43.6±1.3	40.9±5.5
QRS duration (msec)	18.6±0.5	19.3±1.5
QT duration (msec)	51.7±1.3	50.9±1.3
Transesophageal recording and fixed rate pacing		
WP (msec)	77.8±2.0	76.9±3.6
SNRT (msec)	178.4±18.6	183.3±8.7
SACT (msec)	22.3±1.9	22.4±1.7
Programmed atrial stimulation		
AVNRP (S1S2), S1S1 120 msec	70.5±0.9	65.2±2.0^a^
AVNRP (S1S2), S1S1 110 msec	69.5±0.93	65.1±1.9^a^
AVNRP (S1S2), S1S1 100 msec	69.25±1.0	64.4±1.8^a^
ARP (S1S2), S1S1 120 msec	58.5±2.4	48.3±2.7^a^
ARP (S1S2), S1S1 110 msec	56.8±2.3	50.1±3.1^a^
ARP (S1S2), S1S1 100 msec	56.5±2.0	46.4±3.8^a^

a P<0.05 vs. Sham. Sham, sham-operated rats; SP, rats with sterile pericarditis; ECG, electrocardiogram; SCL, sinus cycle length; WP, Wenckebach periodicity; SNRT, sinus node recovery time; SACT, rate corrected SNRT; AVNRP, atrioventricular (AV) nodal refractory period; ARP, atrial nodal refractory period.
